# Tissue-engineered liver using 3D-printed silk fibroin scaffolds loaded with stem cells for the treatment of acute liver injury

**DOI:** 10.1093/rb/rbaf103

**Published:** 2025-10-23

**Authors:** Xiaonan Shi, Xuan Wu, Daxu Zhang, Feng Du, Jingjing Hu, Zhanbo Wang, Yutong Liu, Sanduo Li, Shuo Zhao, Weilong Li, Shujun Ye, Jingyi Wang, Xiaojiao Liu, Yaopeng Zhang, Li Yan

**Affiliations:** The Second Medical Center and National Clinical Research Center of Geriatric Diseases, Chinese PLA General Hospital, Beijing 100853, PR China; The Second Medical Center and National Clinical Research Center of Geriatric Diseases, Chinese PLA General Hospital, Beijing 100853, PR China; Faculty and Institute of Hepato-Pancreato-Biliary Surgery, First Medical Center, Chinese PLA General Hospital, Beijing 100853, PR China; Department of Gastroenterology, Beijing Friendship Hospital, Capital Medical University, State Key Laboratory of Digestive Health, National Clinical Research Center for Digestive Diseases, Beijing Key Laboratory of Early Gastrointestinal Cancer Medicine and Medical Devices, Beijing 100050, PR China; The Second Medical Center and National Clinical Research Center of Geriatric Diseases, Chinese PLA General Hospital, Beijing 100853, PR China; Department of Pathology, Chinese PLA General Hospital, Beijing, 100853, PR China; The Second Medical Center and National Clinical Research Center of Geriatric Diseases, Chinese PLA General Hospital, Beijing 100853, PR China; School of Public Health, Zhejiang Chinese Medical University, Hangzhou 310053, PR China; The Second Medical Center and National Clinical Research Center of Geriatric Diseases, Chinese PLA General Hospital, Beijing 100853, PR China; The Second Medical Center and National Clinical Research Center of Geriatric Diseases, Chinese PLA General Hospital, Beijing 100853, PR China; The Second Medical Center and National Clinical Research Center of Geriatric Diseases, Chinese PLA General Hospital, Beijing 100853, PR China; The Second Medical Center and National Clinical Research Center of Geriatric Diseases, Chinese PLA General Hospital, Beijing 100853, PR China; State Key Laboratory for Modification of Chemical Fibers and Polymer Materials, College of Materials Science and Engineering, Shanghai Engineering Research Center of Nano-Biomaterials and Regenerative Medicine, Donghua University, Shanghai 201620, PR China; State Key Laboratory for Modification of Chemical Fibers and Polymer Materials, College of Materials Science and Engineering, Shanghai Engineering Research Center of Nano-Biomaterials and Regenerative Medicine, Donghua University, Shanghai 201620, PR China; The Second Medical Center and National Clinical Research Center of Geriatric Diseases, Chinese PLA General Hospital, Beijing 100853, PR China

**Keywords:** tissue engineering, adipose-derived stem cells, acute liver injury, liver tissue engineering, 3D printing

## Abstract

Liver tissue engineering offers a promising therapeutic strategy for acute liver injury (ALI). Although traditional biomaterial scaffolds exhibit favorable biocompatibility, they still face limitations in the construction of precise structures and the design of functional properties, making it difficult to fully meet the requirements for the repair of specific organs and tissues. In recent years, 3D-printed silk fibroin (3D-SF) scaffolds have demonstrated broad application prospects in tissue and organ repair owing to their excellent biological properties. In this study, a silk fibroin (SF) solution was used as bioink to successfully fabricate 3D-SF scaffolds with fine microarchitectures and mechanical properties matching those of ALI-affected liver tissue, employing a 4K-resolution micro-nano 3D printer integrated with digital light processing technology. *In vitro* results demonstrated that adipose-derived mesenchymal stem cells (ADSCs) were able to adhere, proliferate and differentiate into hepatocyte-like cells within the 3D-SF scaffolds under specific inductive factors. *In vivo*, after transplanting 3D-SF onto the liver surface of ALI mice, liver function was partially improved and hepatic injury was repaired. The combination of ADSCs and 3D-SF (ADSCs@3D-SF) significantly enhanced the efficiency of ALI repair. Pathological analysis revealed the formation of vascular and biliary duct-like structures at the scaffold–liver interface. Transcriptomic analysis further indicated that ADSCs@3D-SF upregulated the mRNA and protein expression levels of β-Catenin, LEF1 and Cyclin D1 in the Wnt signaling pathway, promoting cell proliferation and facilitating the recovery from ALI. These findings suggest that ADSCs@3D-SF hold promise as a scaffold candidate for liver tissue engineering, offering a novel strategy for the treatment of liver diseases and the reconstruction of vascular systems.

## Graphical Abstract

**Figure:**
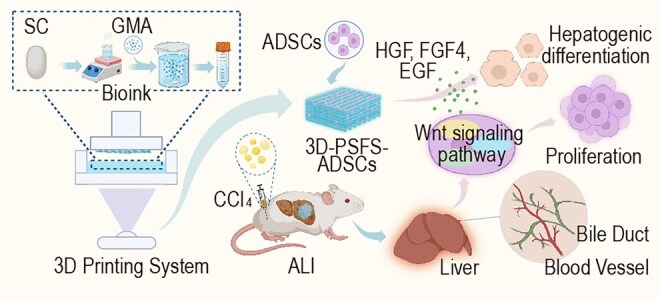
The Graphical Abstract was created using Biorender, and the publication license has been uploaded.

## Introduction

Acute liver injury (ALI) is characterized by a rapid loss of liver function, most commonly caused by alcohol, drugs and infections. Once ALI progresses to liver failure, the mortality rate can reach as high as 80–85% [1]. In clinical practice, liver transplantation is constrained by a shortage of donor organs and prohibitive costs. Bioartificial liver systems provide only short-term, partial functional support and fail to fundamentally restore hepatic tissue. Similarly, stem cell infusion therapy faces challenges such as poor engraftment efficiency within the injured liver and the risk of ectopic embolization. These limitations underscore the urgent need for novel therapeutic strategies for ALI [2–4].

In recent years, liver tissue engineering, which integrates stem cell differentiation with functionalized biomaterial scaffolds, has emerged as one of the most promising approaches for ALI therapy. By recapitulating the hepatic microenvironment and delivering stem cells via biomaterial scaffolds for localized transplantation, this strategy enhances stem cell engraftment within the injured liver, effectively reduces the risk of ectopic embolization and enables both transdifferentiation and paracrine effects. Collectively, these advantages improve therapeutic safety and clinical efficacy [5].

To date, human embryonic stem cells, induced pluripotent stem cells [6], bone marrow-derived mesenchymal stem cells (MSCs) and ADSCs have all been applied in liver tissue engineering [7]. Among these, ADSCs have shown particular promise in liver regeneration owing to their anti-inflammatory, anti-apoptotic and pro-angiogenic properties [8, 9]. Previous studies have demonstrated that ADSCs combined with plant fiber scaffolds, electrospun protein fiber scaffolds or decellularized liver scaffolds can improve liver function in ALI mouse models [10–12]. Nevertheless, these materials still present limitations, including complex preparation processes, insufficient mechanical strength and restricted microstructural tunability [13].

Through 3D printing technology, complex 3D structures can be precisely constructed, thereby better simulating the hepatic microarchitecture [14]. However, conventional 3D bioprinting approaches face limitations such as low printing accuracy, low efficiency, complex processes and high costs [15]. In the fabrication of complex hepatic structures, additional challenges include difficulties in accurately controlling scaffold pore size, incomplete construction of nutrient transport channels and insufficient compatibility with highly biocompatible materials [16, 17]. A variety of polymeric materials, such as sodium alginate hydrogel, poly lactic-co-glycolic acid, polylactic acid (PLA), polycaprolactone and silk fibroin (SF), have been widely used in diverse fields of tissue engineering [18–20]. Among them, SF has attracted considerable attention owing to its excellent biocompatibility, mechanical strength and chemical modifiability [21, 22]. Nevertheless, the application of 3D-printed silk fibroin (3D-SF) scaffolds in combination with stem cells for liver tissue engineering has not yet been systematically investigated.

In this study, we employed high-resolution digital light processing (DLP) printing technology (3840 × 2160 resolution, optical accuracy of 5 µm) to fabricate 3D-SF scaffolds. Each layer of the computer-aided design (CAD) model was projected into the SF bioink via ultraviolet irradiation, where photocrosslinking occurred sequentially to form the 3D scaffold structure. The microarchitecture of the 3D-SF scaffolds was subsequently observed using scanning electron microscopy (SEM). *In vitro* experiments were conducted to assess the biocompatibility of 3D-SF with ADSCs as well as the hepatogenic differentiation potential of ADSCs under the stimulation of liver-inducing factors. For *in vivo* studies, ADSCs@3D-SF constructs were transplanted onto the liver surface of ALI mice, and serum biochemical tests along with histopathological analyses were performed to evaluate liver function recovery and tissue repair. In addition, immunohistochemistry, transcriptomics, real-time quantitative PCR (RT-qPCR) and western blotting (WB) were applied to elucidate the molecular mechanisms underlying liver regeneration. This study aims to provide a biomaterial scaffold with high-precision microstructures, favorable mechanical properties and excellent biocompatibility for liver tissue engineering, as well as to offer a novel strategy for the treatment of ALI.

## Materials and methods

### Animals

All animal experiments were approved by the Institutional Animal Care and Use Committee of the Chinese PLA General Hospital. The animal testing license code is 2019YFA010600. ADSCs were separated from 3-day-old BALB/c mice (SPF Biotechnology Co., China). Healthy male BALB/c mice of 6–8 weeks old (SPF Biotechnology Co., China) were used to evaluate the therapeutic effect of ADSCs@3D-SF. The method for constructing ALI mouse model has been previously described in our study [23]. Briefly, the ALI model was prepared by injected CCl_4_ (100 µL olive oil per 10 µL CCl_4_) for 20 g of body weight intraperitoneally.

### Preparation of 3D printing bioink

The SC used in this study was obtained from Hangzhou, China. SC (40 g) was degummed by boiling in Na_2_CO_3_ (1 liter, 0.05 M, Macklin, USA) solution for 1 h. The SF was rinsed 2 or 3 times with distilled water and dehydrated overnight in a circulating oven. Then, SF (20 g) was dissolved in LiBr (100 mL, 9.3 M, Macklin) at 60°C for 1 h, and filtered through a filter (100 μm, FALCON). After glycidyl methacrylate (GMA) (6 mL, 424 mM, YuanYe Bio-Technology) was slowly added into the SF, the SF solution with GMA was deposited on an agitator and stirred at 300 rpms at 60°C for 3 h to complete the grafting reaction between SF and GMA. Then, the SF modified by GMA (Sil-MA) solution was filtered through a filter (100 μm) and dialyzed in distilled water for 4 days using a dialysis membrane (12–14 kDa, Viskase). Subsequently, the methacrylated SF solutions were lyophilized using a freezer for 48 h. The Sil-MA powder was dissolved in distilled water and stirred until fully dissolved to prepare a Sil-MA solution (30% w/v). Then, lithium phenyl-2,4,6-trimethylbenzoylphosphinate (LAP, Innochem) was dissolved in PBS (Gibco) to make a LAP solution (0.2% w/v), which was then added to the Sil-MA solution to prepare the 3D printing bioink.

### 3D printed scaffolds preparation

3D printing was performed by using a 4K resolution micro-nano 3D printer (Shenzhen Guangyunda Optoelectronics, China) with a resolution of 3840 × 2160 and an optical precision of 5 μm. The operation procedure is as follows: first, the bioink is placed in the material tank; the optical engine projects exposure patterns from below, transmitting the patterns through the transparent release film at the bottom of the tank. After photocuring, the SF adheres to the forming plate. As the forming plate gradually rises, the optical engine switches exposure patterns, achieving the photocuring and printing of the 3D structure of the SF. Briefly, parameters for 3D printing were following exposure wavelength was 405 nm, the light power density was 21 mW/cm^2^ and each exposure time was 0.8 s. Each cured layer had a thickness of 50 μm. It should be noted that the single-layer thickness of the cell scaffold model was 100 μm. To avoid inconsistencies in the thickness of the horizontal and vertical bars of the printed cell scaffold, the single-layer thickness of the cell scaffold model should be an integer multiple of the photocured layer thickness. After printing, the 3D-SF was soaked in a 100°C water bath to remove any uncured SF solution. Subsequently, the scaffold was subjected to a drying process.

### Fourier transform infrared spectroscopy assay

Mounted the polarized Universal Attenuated Total Reflection (UATR) device on an optical bench in Frontier IR/NIR system. The front beam splitter detector switching system automatically detected the UATR device, effect settings and range switching.

Subsequently, Sil-MA (2 mg) sponge and SF were ground with KBr (300 mg) in the agate mortar and pestle to complete mixing. Finally, launched the Spectrum 10 software suite on computer. A small amount of the material was placed on the Fourier transform infrared spectroscopy (FT-IR) spectrometer detector using tweezers. The removable light beam was positioned over the material. Then, readings were initiated using the Spectrum software suite. Due to the interaction of infrared light in samples, the molecules of samples underwent vibrational motion, resulting in an infrared spectrum with multiple peaks. Based on the intensity (weak, medium, strong), shape (broad or sharp) and position (cm^−1^), the characteristics of the sample peaks were compared with SF. The observed changes in peak characteristics were attributed to the methacrylation of SF.

### Proton nuclear magnetic resonance assay

To determine the degree of methacrylation (DoM), proton nuclear magnetic resonance (^1^H-NMR) spectroscopy was performed using a Bruker 600 MHz AVANCE III HD. Sil-MA samples (2.5 mg) were dissolved in 500 μL deuterium oxide (D_2_O, Sigma-Aldrich) and filtered through a 0.45 μm membrane prior to measurement. ^1^H-NMR spectra of both SF and Sil-MA were recorded. The DoM was defined as the percentage of ε-amino groups of lysine in SF modified after methacrylation. The lysine methylene protons at δ = 2.83 ppm were integrated to calculate the relative decrease. The degree of substitution was subsequently determined using the following formula:


$$\text{DoM}\;(\%)=1-(\frac{\text{Lysineintegratedsignal}\;\text{of}\;\text{functionalized}\;\text{Sil-MA}}{\text{Lysineintegratedsignal}\;\text{of}\;\text{SF}}\times 100\%)$$

### Compressive elastic modulus assay

Stress–strain tests were measured using a universal testing machine (MTSc42.503y). Samples (*L* = 1 cm, *W* = 1 cm, *H* = 1.6 mm) were placed on the testing platform and a compressive load was applied at a displacement rate of 5 mm/min until failure. The values of stress (Pa) and strain (%) were obtained from the stress–strain curve of the failure point. Finally, we calculated the compressive elastic modulus of the 3D-SF according to the following formula,


$$\text{Ec}=\frac{\sigma }{\epsilon }$$

where Ec is compressive elastic modulus (kPa), σ is compressive stress and ϵ is compressive strain.

### Water absorption test assay

After freezing the 3D-SF samples, we weighed them to obtain the initial weight (W1) and measure the diameter and thickness of the samples. The dried samples were then incubated in ultrapure water at room temperature. At specified time intervals (0, 1, 2, 3, 5, 10, 15, 30 min, 1 h), collected the samples and weighed them to obtain the swollen weight (W2), and measured the diameter and thickness of the hydrogels. The swelling ratio (SR) of the scaffold samples can be calculated using the following equation.


SR = (W2 − W1)/W2 × 100%

### Degradation test *in vitro*

The 3D-SF scaffolds were incubated in 5 ml of PBS containing 2 U of collagenase II and harvested at predetermined time points (0, 2, 4, 8, 12 and 24 h), followed by freeze-drying. The control group was incubated in 5 ml of PBS without enzyme. The dry weight of each scaffold after freeze-drying was recorded as *W*d (*W*_d0_ for 0 h and *W*_dn_ for subsequent time points). The *in vitro* degradation ratio (DR) was calculated using the following equation[24]:


$$\text{DR}\;\text{in}\;\text{vitro}=\frac{\;W_{d0}-W_{\text{dn}}}{\;W_{d0}}\times 100\%$$

### Cell culture assay

ADSCs were purified from 3-day-old BALB/c neonatal mice. First, the mice were euthanized and disinfected, and the inguinal adipose tissue was isolated. The adipose tissue was minced and incubated with type I collagenase (1 mg/mL), thoroughly mixed and centrifuged. The lower layer of cells was collected and seeded into cell culture flasks to obtain primary ADSCs. Prior to cell seeding, the 3D-SF were sterilized by cobalt-60 radiation, placed in 24-well plates and immersed in sterile water at 100°C for 20 min in a biosafety cabinet. Subsequently, ADSCs from T75 culture flasks (Corning, USA) were digested, centrifuged and resuspended in MEM-F12 medium (Gibco, USA) containing 10% fetal bovine serum (FBS, Gibco, USA) to a cell density of 10^6^ cells/ml, and then seeded onto the scaffolds. The MEM-F12 medium was changed every other day.

### Cell induction

The sterilized bioscaffolds were placed in 24-well culture plates (Corning, USA). Then, 1 × 10^6^ ADSCs were seeded into each 3D-SF. The ADSCs were cultured at 37°C with 5% CO_2_. Once the ADSCs adhered to 3D-SF, basic medium (MEM-F12 with 10% FBS) was replaced with hepatocyte induction medium which has been used in our previous research [10]. Then, 1.5 ml of hepatocyte induction medium was added to each well of the 24-well culture plate and replaced every 48 h.

### SEM assay

On the third day of culture, 3D-SF and ADSCs@3D-SF were collected. The specimens were rinsed with PBS 2–3 times. Then, samples were fixed in 2.5% glutaraldehyde solution for 4 h. Subsequently, the samples were subjected to gradient dehydration with different concentrations of ethanol (50%, 60%, 70%, 80%, 90%, 100%). Each concentration was treated for 10 min and repeated twice. The samples were then replaced with isoamyl acetate for 40 min, followed by immersion in hexamethyldisilazane for 8–10 min. After critical point drying and spray gold treatment, the morphology and porosity of 3D-SF were observed by SEM (S-4800, Hitachi, Japan). Images were analyzed by Image J software.

### Detection of cell viability

Cell viability within the 3D-SF was assessed on days 1, 3 and 5 of culture. Samples were stained with 5-chloromethylfluorescein diacetate-propidium iodide (CMFDA-PI) using the Live/Dead Cell Viability Assay Kit (Invitrogen, Carlsbad, CA, USA) and observed under a confocal fluorescence microscope (A1/LSM-Kit). Living cells emit green fluorescence, while dead cells emit red fluorescence. Each sample randomly selected three different regions for quantitative analysis. Image J software was used to classify and count the colors in the images. The calculation of cell viability can be determined by utilizing the formula provided below.


$$\text{Cell}\;\text{viability}\;(\%)=\frac{\text{Number}\;\text{of}\;\text{live}\;\text{cells}}{\text{Number}\;\text{of}\;\text{live}\;\text{cells}+\text{Number}\;\text{of}\;\text{dead}\;\text{cells}\;}\times 100\%$$

Additionally, confocal fluorescence microscopy panoramic scanning (vertical interval of 200 μm) was performed to create multi-layer composite images, allowing for the observation of overall cell distribution and changes in cell density within the samples.

### Cell proliferation assay

The proliferation of ADSCs cultured on 3D-SF on the first, third and fifth day could be quantitatively evaluated by CCK-8 kit (Tokyo, Japan). According to the steps of reagent use, ADSCs were incubated with CCK-8 working solution at 37°C for 2 h, and the absorbance of the solution was detected by a microplate reader (Beckman, Fullerton, CA, USA).

### Immunofluorescence assay of liver-like cells

Before immunofluorescence staining, ADSCs@3D-SF should be fixed with 2.5% paraformaldehyde (PFA) and frozen in 30% sucrose. The samples were immediately embedded in cryosectioning medium (Leica) and dehydrated. Then, they were sectioned into 10 μm slices by the cryostat (CM1950, Leica). The frozen sections were fixed with 4% PFA after rehydration. After blocking with PBS (5% goat serum and 2% bovine serum albumin), the following primary antibodies were added to the frozen sections and incubated overnight at 4°C: Cytochrome P450 (CYP1A1, ab235185, Abcam), Cytokeratin 18 (CK18, D4G2, CST), Alpha-Fetoprotein (AFP, ab213328, Abcam) and Albumin (ALB, ab207327, Abcam). Subsequently, secondary antibodies were added to the sections and incubated for 1 h at room temperature. Besides, the nucleus was restained with 4′,6-diamidino-2-phenylindole (DAPI). For negative controls, an equal amount of Ig G from normal mouse (Vector Labs, Buringame, CA, USA) was used instead of the primary antibody for immunofluorescence staining, Finally, images were collected by a fluorescence microscope (Nikon, Japan).

### ELISA

To verify that ADSCs can be induced to be hepatocyte-like cells more effectively within the 3D-SF, supernatants were collected on days 1, 3, 5, 7 and 14 of cell culture. According to the kit instructions, the concentrations of AFP and ALB were determined by using the corresponding ELISA kit (Beijing Chenglin Biotechnology Co., Ltd, China).

### Real-time quantitative polymerase chain reaction analysis

To verify the mRNA expression of target genes including ALB, AFP, CK18, Cytokeratin-19 (CK19, ab76539, Abcam), CYP1A1, β-Catenin (ab32572, Abcam), LEF1 (ab137872, Abcam) and Cyclin D1 (55506 T, CST) in ADSCs (primer sequences are shown in Figure S1), total RNA was extracted using the Trizol (Invitrogen Life Technologies) method. Determine the purity and concentration of RNA. cDNA was synthesized from RNA by reverse transcription under the following conditions: 50°C for 15 min, 85°C for 5 s. Synthesized cDNA should be stored in a 4°C refrigerator. Subsequently, RT-qPCR was performed according to the instructions: 95°C for 30 s (initial denaturation), 40 cycles of 95°C for 10 s (annealing), 60°C for 30 s (extension). Each reaction was run in triplicate, with ACTIN used as internal control. The relative expression levels of mRNA were calculated by 2^−ΔΔCt^ method.

### Western-blot analysis

Western-blot analysis was conducted using the bicinchoninic acid method to prepare and quantify total cellular protein. The lysate solution (80 µg) was separated on a sodium dodecyl sulfate-polyacrylamide gel electrophoresis (SDS–PAGE) gel (10%) for 60 min at 80–120 V. Subsequently, transferred it to the nitrocellulose membrane (Immoblin-P, Millipore, Bedford, MA, USA). After closuring, the membrane with 5% skimmed milk powder at room temperature for 2 h, the primary antibody was incubated overnight at 4°C, including rabbit anti-mouse ALB, AFP, CK18, CK19, CYP1A1, β-Catenin, LEF1 and Cyclin D1 (1 : 1000, ZEN-BIOSCIENCE). Tris-buffer saline with Tween 20 (TBST) was used to wash the samples 3 times, 15 min each time. Subsequently, the membrane was incubated with Horseradish Peroxidase (HRP) conjugated secondary antibodies (goat anti-rabbit Ig G, HRP-labeled goat anti-mouse Ig G, Beijing Zhongshan Golden Bridge Biotechnology, China) at room temperature for 2 h. The membrane was washed again in TBST and added the enhanced chemiluminescence reagents. GAPDH was used as the internal reference. The Image J software was used to analyze the gray values of the target protein bands.

### Transplantation and sample collection

All surgeries were performed by the same surgeon. Pentobarbital (1%, 50 mg/kg) was injected intraperitoneally for anesthesia. The liver’s left lateral lobe was exposed, and 3D-SF was sutured in place. The wounds were closed in layers. Liver tissue and blood were collected on days of 2, 5, 7 and 14 post-transplantations for subsequent experiments. *n* = 3 mice per group.

### Liver function and C-reactive protein evaluation

On days 2, 5, 7 and 14 post-transplantation, serum levels of Alanine Aminotransferase (ALT), Aspartate Transferase (AST), Alkaline Phosphatase (ALP), ALB, Total Bilirubin (TBIL), Triglycerides (TG), Total Protein (TP) and C-reactive protein (CRP) were measured using an automated analyzer (Mindray, BS-240 Vet).

### Histological staining and evaluation

The collected liver tissues were fixed in 4% PFA for 2 days and then the paraffin was used to embed tissues. The paraffin blocks were sectioned into 6 µm slices using the microtome (Leica SM2000R). Hematoxylin and eosin staining, and histological scoring were performed according to instructions (Solarbio, *n* = 3).

### Immunohistochemical staining

Frozen liver tissues were sectioned and rehydrated, followed by fixation with 4% PFA. Endogenous peroxidase activity was blocked, and tissue sections were then blocked with PBS (5% goat serum, 2% BSA). Subsequently, the following primary antibodies were added to the sections and incubated overnight at 4°C: anti-CD34 (CD34, Abcam), anti-ERG (ERG, Abcam), anti-MUC-1 (MUC 1, Abcam), anti-ARG-1 (ARG 1, Abcam) and CK19. Afterward, the second antibody was added to the slice and incubated at room temperature for 1 h, then incubated with 3,3′-diaminobenzidine substrate and counterstaining with hematoxylin. The end, slides were coverslipped with neutral resin, and images were collected using the microscope (Nikon, Japan). *n* = 3 samples in each group.

### mRNA transcriptome sequencing analysis

We performed mRNA transcriptomic sequencing to analyze mRNA changes in the 3D-SF group and ADSCs@3D-SF group during the fifth day of liver repair. First, mouse liver tissues were dissolved using Trizol Reagen for total RNA extraction. Subsequently, the purity and concentration of RNA were evaluated by NanoDrop spectrophotometer (Thermo Scientific), and the integrity and quantity of RNA were evaluated by Agilent 2100/4200 system. Next, a sequencing library was prepared from 3 µg of RNA. Initially, mRNA could enrich from the total RNA by oligo-conjugated magnetic beads in a specific buffer, followed by fragmentation of mRNA using divalent cations. Subsequently, random hexamers were used to synthesize the first cDNA strand. The buffer, dNTPs, RNaseH and DNA polymerase I were used to synthesize the second cDNA strand. Poly-A tails were added to the 3′ ends of cDNA to repair double-stranded DNA. The product was purified and fragmented using Hieff NGS @ DNA selective magnetic beads for PCR amplification and enrichment. Sequencing libraries were prepared using the PE150 model from Berry Genomics Co., Ltd (Wuhan, China) on DNBSEO-T7, and were sequenced after passing quality control checks. Finally, differential gene expression analysis was conducted according to the sequencing results, and significant enrichment analysis of differentially expressed genes was performed using KEGG pathways.

### Statistical analysis

For statistical analysis, the Student’s *t*-test was employed to compare parameter data between two groups. The normality of the data was verified, and all analyses were carried out using Graph Pad Prism 9 software, developed by Graph Pad Software Inc. in San Diego, CA, USA. The findings are expressed as mean values with SD. The significance level of *P* < 0.05 was considered statistically significant.

## Results and discussion

### Synthesis of Sil-MA

**Figure 1.**
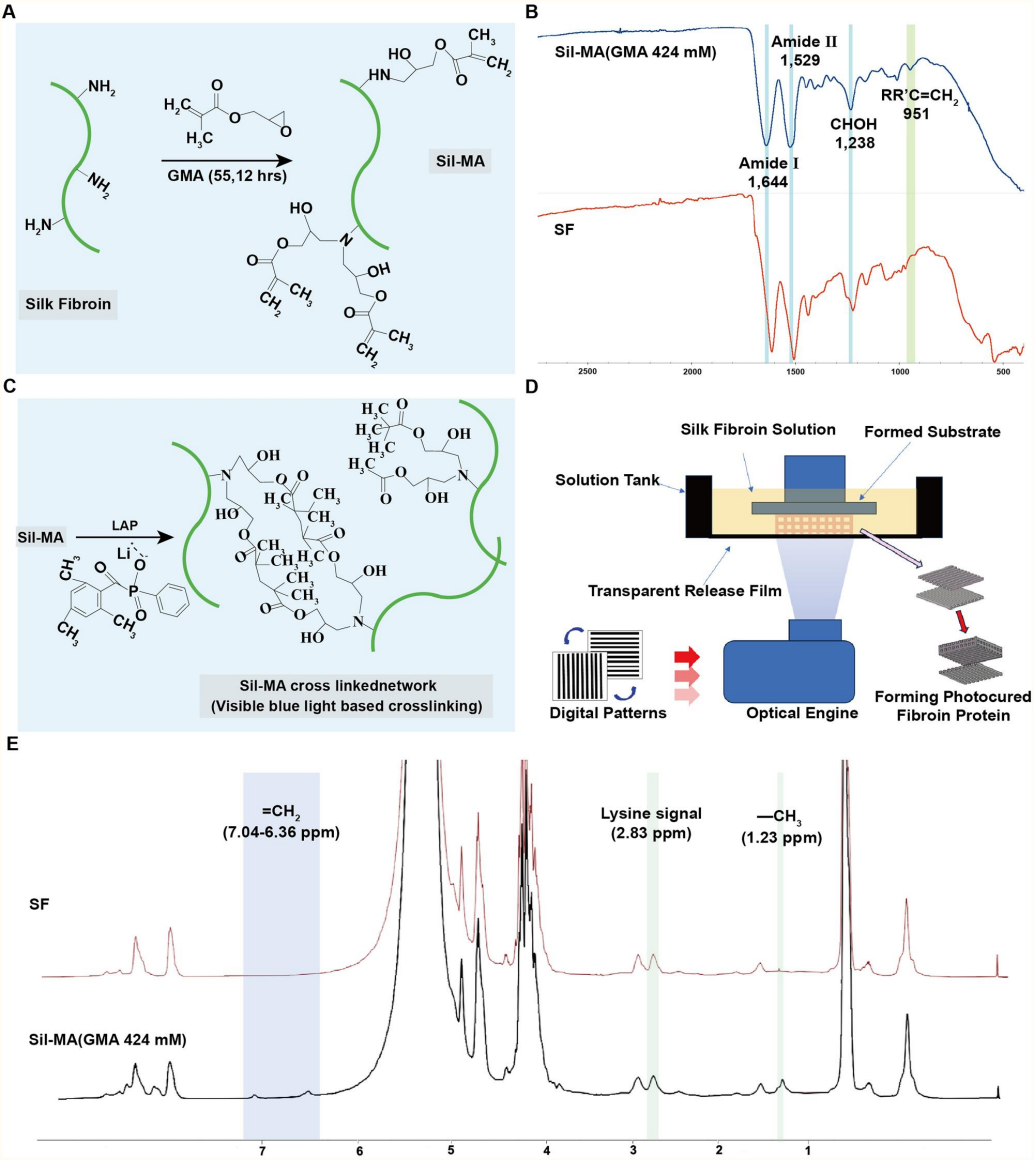
Synthesis of Sil-MA. (**A**) Schematic diagram of SF and GMA grafting reactions. (**B**) FT-IR plots of SF before and after grafting with GMA. (**C**) Schematic diagram of Sil-MA and LAP light curing reactions. (**D**) Flowchart of the printing process of 3D-SF. (**E**) ^1^H-NMR spectra of SF and Sil-MA [25].

To prepare bio-ink, GMA was used to produce Sil-MA by grafting. Briefly, SF coupled with the active epoxy groups from GMA, completes the reaction to produce Sil-MA (Figure 1A). By FT-IR assay, the spectra of pre- and post-grafting SF showed the characteristic peaks of Sil-MA at 1644 cm^−1^, 1529 cm^−1^ corresponding to amide I and II bands, respectively. The peak at 1238 cm^−1^ indicated the conversion of epoxy groups in GMA to hydroxyl groups (CHOH stretching). Additionally, a peak at 951 cm^−1^ corresponded to the CH_2_ wagging vibration of the ethylene group in methacrylate ester, confirming successful incorporation of GMA into SF via covalent bonding (Figure 1B). Upon addition of LAP to Sil-MA, the ethylene double bonds on GMA could undergo intra- or inter-chain reactions (Figure 1C). Using a 405 nm UV light, modulated into digital patterns through a polarization modulator illuminated by a uniform light system, images were projected onto the SF bio-ink, enabling the 3D-SF (Figure 1D). The DoM was determined by ^1^H-NMR spectroscopy (Figure 1E). The expected ring-opening of the GMA epoxy group and subsequent nucleophilic addition of lysine residues were confirmed by characteristic spectral changes. Specifically, new resonances corresponding to the methacrylate vinyl protons (δ  =  7.04 – 6.38 ppm) and the GMA methyl protons (δ  =  1.23 ppm) appeared upon GMA addition. Concurrently, the lysine methylene signal at δ  =  2.83 ppm decreased, indicating substitution of lysine residues in SF, with a calculated DoM of 58% (Figure S2).

### Characterization of 3D-SF scaffolds

**Figure 2.**
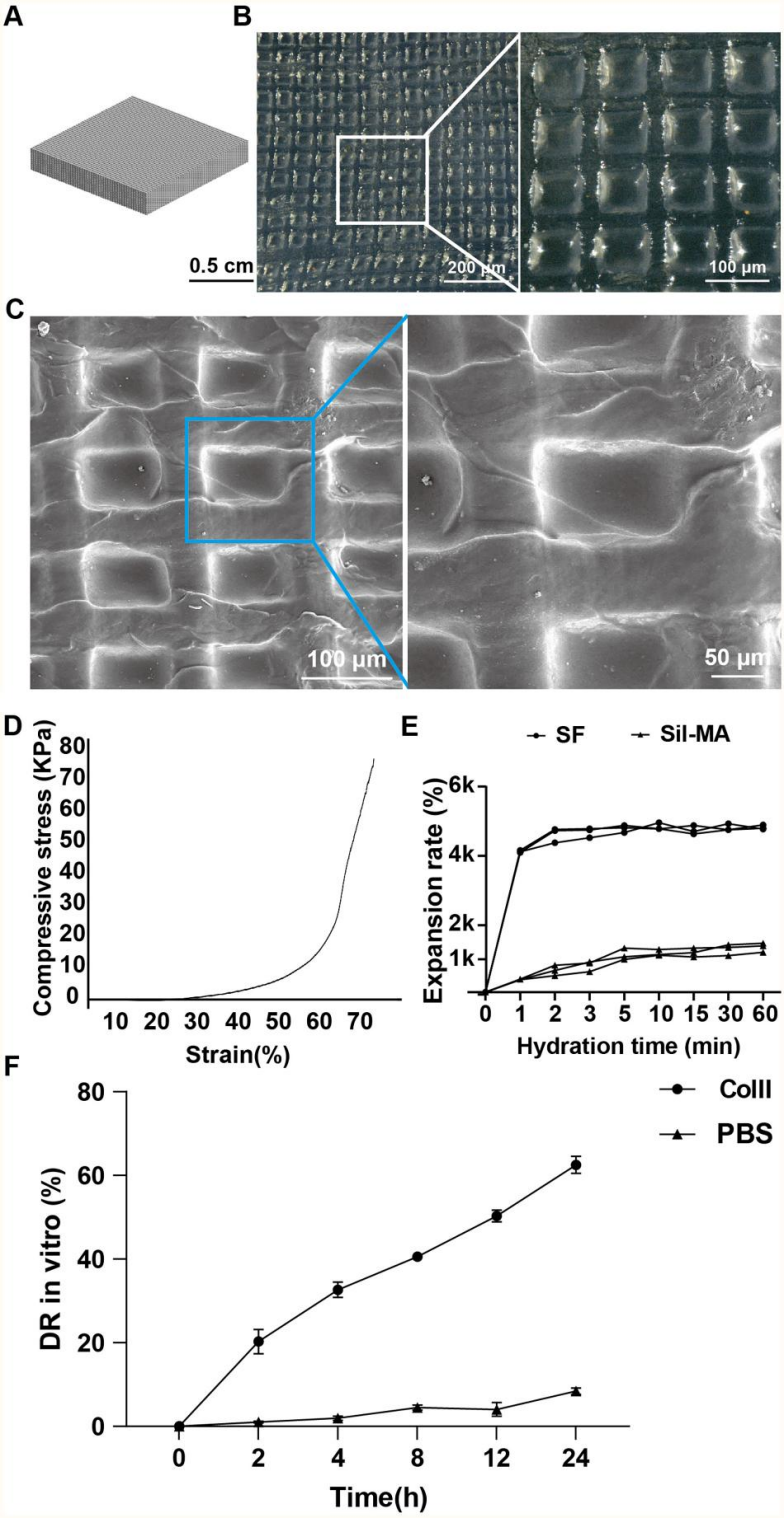
Characterization of 3D-SF. (**A, B**) Overview of 3D-SF. (**C**) SEM images of 3D-SF. (**D**) Compression-strain testing of 3D-SF. (**E**) Water absorption testing of 3D-SF. (**F**) DR of 3D-SF *in vitro* (*n* = 3).

To print high-precision 3D-SF, we designed digital models of scaffolds by CAD methods with length, width of 1.6 mm (Figure S3). The cube with a pore size of 100 μm. The 3D-SF was transparent by microscope observation (Figure 2A). SEM assay showed that 3D-SF were regular in shape, highly porous, with uniformly arranged and sized pores, and the length, width and height were 100 μm. After calculation, the porosity was 44% (Figure 2B). Based on the ratio of stress to deformability, the compressive strain test showed that the scaffold had a maximum compressive modulus of elasticity of 12 kPa (Figure 2C). The water absorption test showed the expansion rate of the GMA-grafted scaffolds increased gradually from the first 5 min to 1 h. SF reached the highest value within the first 1 min and remained unchanged during the subsequent observation time, indicating the distinct different physical properties between SF and Sil-MA (Figure 2D). The degradation of 3D-SF *in vitro* was compared under PBS and collagenase II conditions (Figure 2F). The results demonstrated that the DR in the collagenase group increased markedly over time, reaching ∼60% at 24 h, whereas the PBS control group maintained DRs below 10% throughout the study. These findings indicate that the degradation of 3D-SF is predominantly enzyme-dependent, while the material remains relatively stable in the absence of enzymatic activity.

### Biocompatibility of 3D-SF scaffold materials with ADSCs

**Figure 3.**
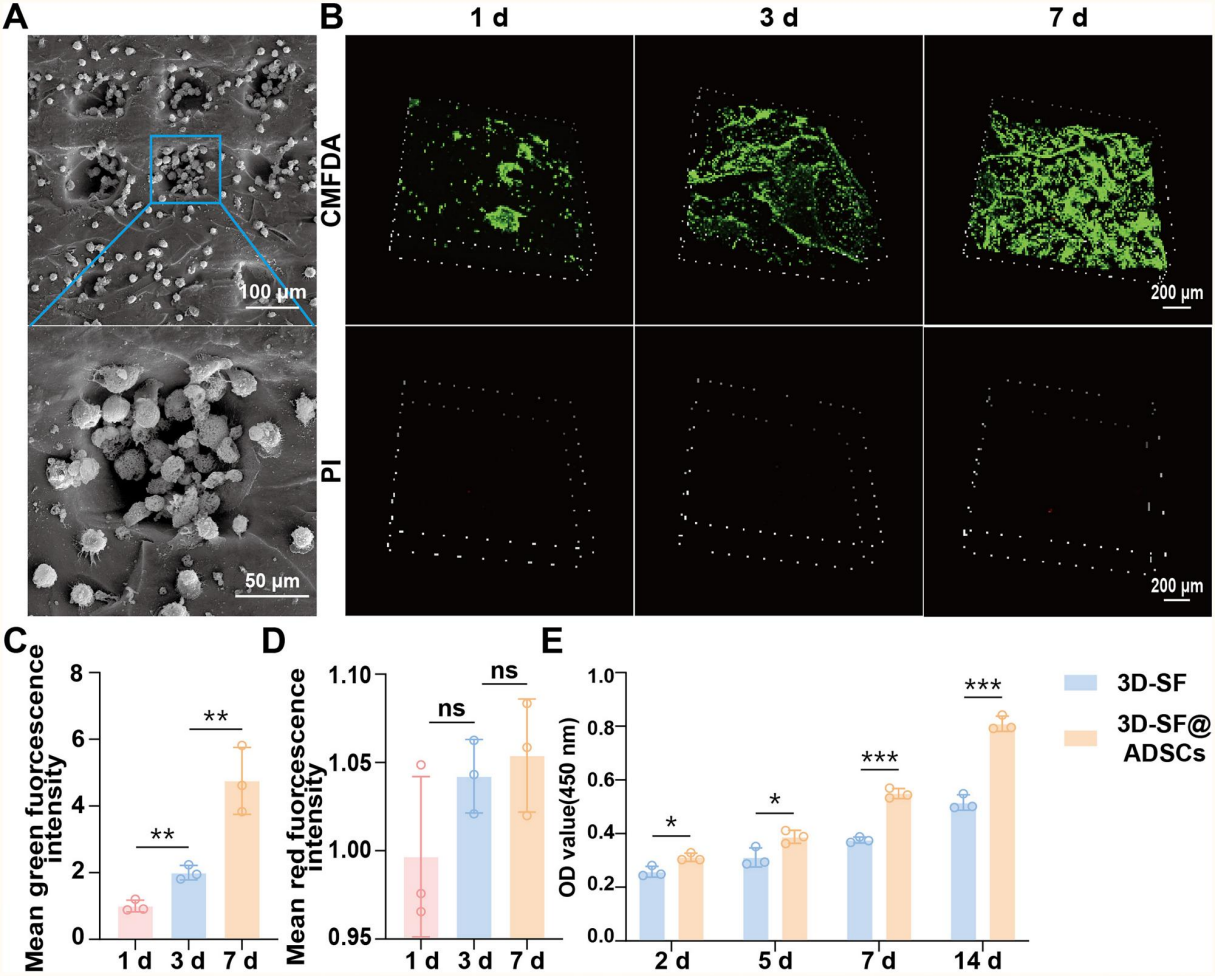
Biocompatibility of ADSCs@3D-SF *in vitro*. (**A**) SEM images of ADSCs adhered to 3D-SF. (**B**) ADSCs cultured in scaffolds were stained alive and dead at 1, 3 and 7 days. (**C**) Green and (**D**) red fluorescence intensities were analyzed by Image J. (**E**) CCK-8 assay showed the significant different proliferation of ADSCs growing between plate and 3D-SF at 2, 5, 7, 14 days. All data are expressed as mean ± SD (*n* = 3), *t*-test statistical analysis: *P < 0.05, **P < 0.01, ***P < 0.001, ns: no significance.

To evaluate the biocompatibility of ADSCs@3D-SF, we seeded ADSCs on 3D-SF to establish an *in vitro* 3D cell culture system. ADSCs adhered to the 3D-SF and could grow in aggregates on the scaffolds with a cell size of 2–3 μm by SEM observation (Figure 3A). Labeled the ADSCs by CMFDA/PI, the results showed that ADSCs grew in 3D-SF and proliferated from 1 to 7 days and were gradually migrated in the scaffolds (Figure 3B). Compared with day 1, the average cell viability in green fluorescence on day 7 was 5-fold higher than that on day 1. Besides, there were only a few dead cells indicated by scattered distribution of red fluorescence during the incubation period (Figure 3C and D). CCK-8 indicated the proliferation of ADSCs in 3D-SF group was obviously increased on days 7 and 14 compared with ADSCs cultured in a conventional 2D environment (Figure 3E). These results indicated that the 3D-SF promoted the attachment and proliferation of ADSCs.

### ADSCs cultured on 3D-SF scaffold material differentiate into hepatocyte-like cells

**Figure 4.**
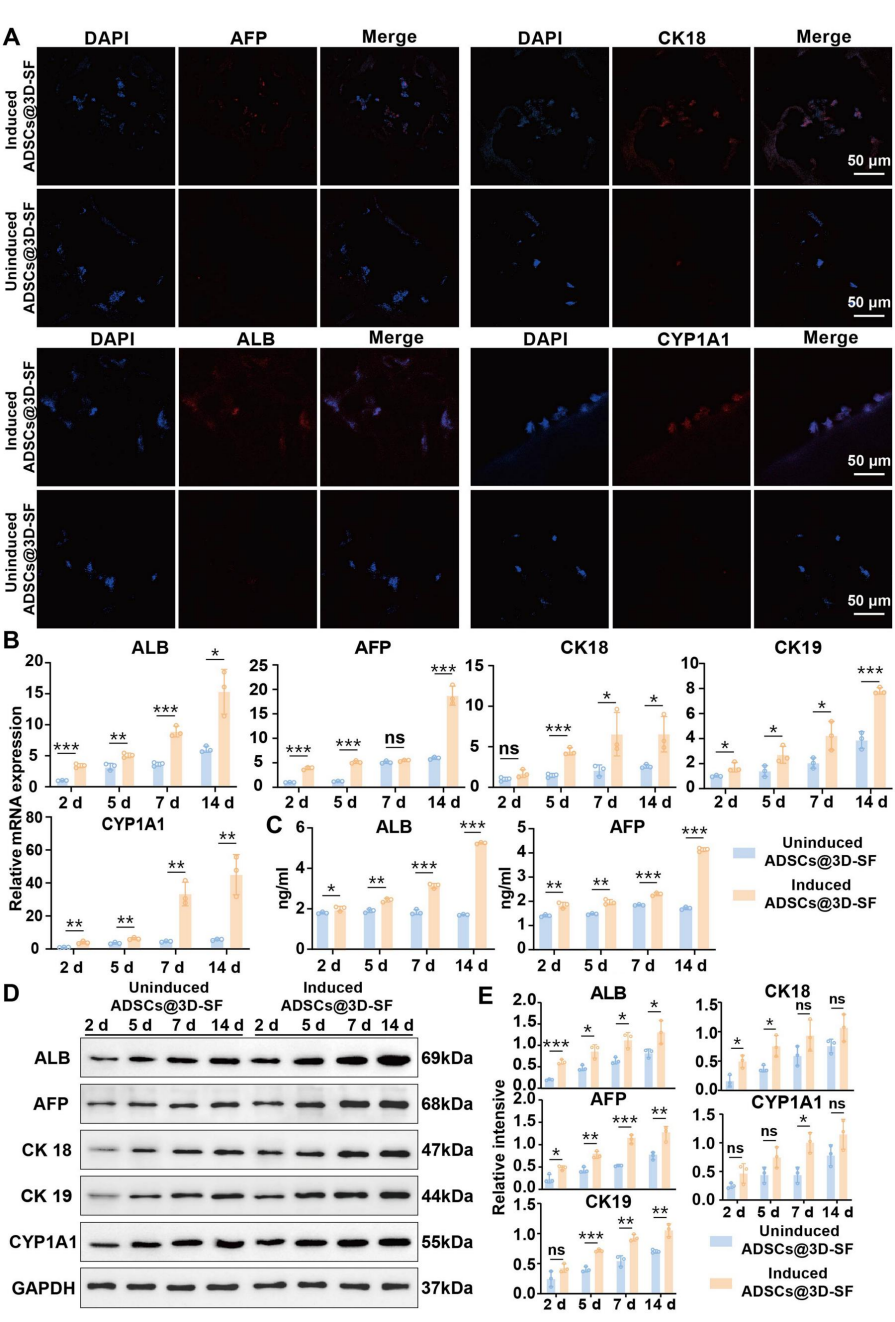
ADSCs Cultured on 3D-SF could differentiate into hepatocyte-like cells *in vitro*. (**A**) Fluorescent microscope was used to analyze the expression levels of liver markers AFP, ALB, CK 18 and CYP 1A1 proteins in 3D-SF hepatocyte-like cells (nuclei: blue, protein: red). (**B**) The RT-qPCR detected the gene expression levels of ALB, AFP, CK 18, CK 19 and CYP 1A1 in ADSCs from control group and 3D-SF group at 2, 5, 7 and 14 days. (**C**) ELISA kit was used to detect the secretion levels of AFP and ALB in cells culture supernatants before and after induction. (**D**) Immunoblot analysis showed the AFP, ALB, CYP1A1, CK 18 and CK 19 protein levels in ADSCs between control and induced group. (**E**) Quantitative analysis of intensities of bands by Image J software to calculate the grayscale value. All data were expressed as mean ± SD (*n* = 3). *t*-Test statistical analysis: *P < 0.05, **P < 0.01, ***P < 0.001, ns: no significance.

The experimental group was ADSCs@3D-SF cultured in an induced environment (hepatocyte induction medium), and the control group was ADSCs@3D-SF cultured in a non-induced environment (basal medium). After 5 days of induced differentiation, we observed that the expression of liver-specific markers such as AFP, ALB, CK18 and CYP1A1 was obviously higher in cells treated with induction than that in the non-induced group by immunofluorescence staining (Figure 4A). The 3D-SF group in a non-induced environment showed that hepatocyte-specific markers were not expressed (Figure S4). On days 2, 5, 7 and 14 after induction, RT-qPCR results showed that the expression levels of AFP, ALB, CK18, CK19 and CYP1A1 in mRNA were significantly increased between ADSCs of induction and that without induction (Figure 4B). Further, the ELISA results indicated that the expression of ALB and AFP in induction-treated cells were obviously higher than that in uninduced cells (Figure 4C). Subsequently, immunoblotting results showed that cells after induction significantly increase the protein level of AFP, ALB, CK18, CK19 and CYP1A1 at various time points compared to the non-induced group (Figure 4D and E). These findings showed that ADSCs cultured on 3D-SF still possess the capability of being induced to differentiate into hepatocyte-like cells.

### Repair of ALI by 3D-SF bioscaffold materials loaded with ADSCs

**Figure 5.**
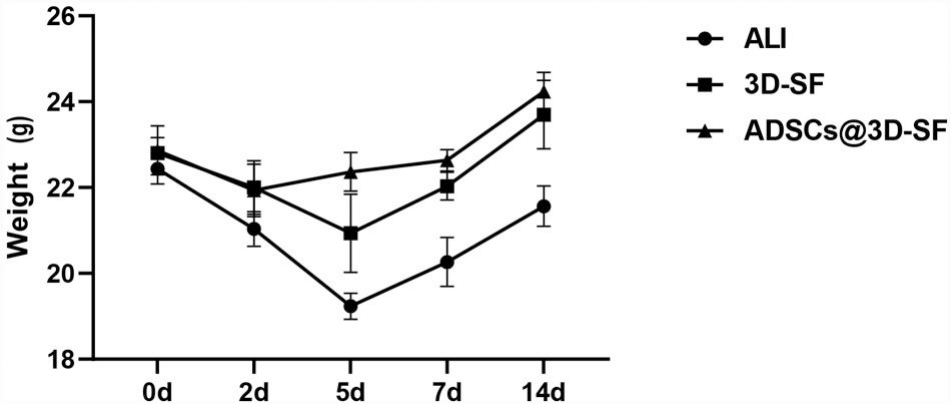
Body weight changes of mice in each group after scaffolds transplantation. (*n* = 3).

The observation time points were 2 weeks after stent transplantation, specifically at days 2, 5, 7 and 14, with 3 mice used for each time point. The average body weight of the mice after modeling was (22.7 ± 0.2) g. In the ALI group, the average body weight decreased to 20.7 g on day 2 after modeling, dropped to 19.5 g on day 5, reached 20.2 g on day 7 and increased to 21.2 g on day 14. In the ADSCs@3D-SF group, the average body weight decreased to 21.9 g on day 2 after transplantation, rose to 22.3 g on day 5, increased to 22.6 g on day 7 and reached 24.2 g on day 14. For the 3D-SF group, the average body weight was 22 g on day 2 after transplantation, decreased to 20.9 g on day 5, rebounded to 22 g on day 7 and increased to 23.7 g on day 14.The trend of body weight changes in the 3D-SF group was similar to that in the ADSCs@3D-SF group (Figure 5).

**Figure 6.**
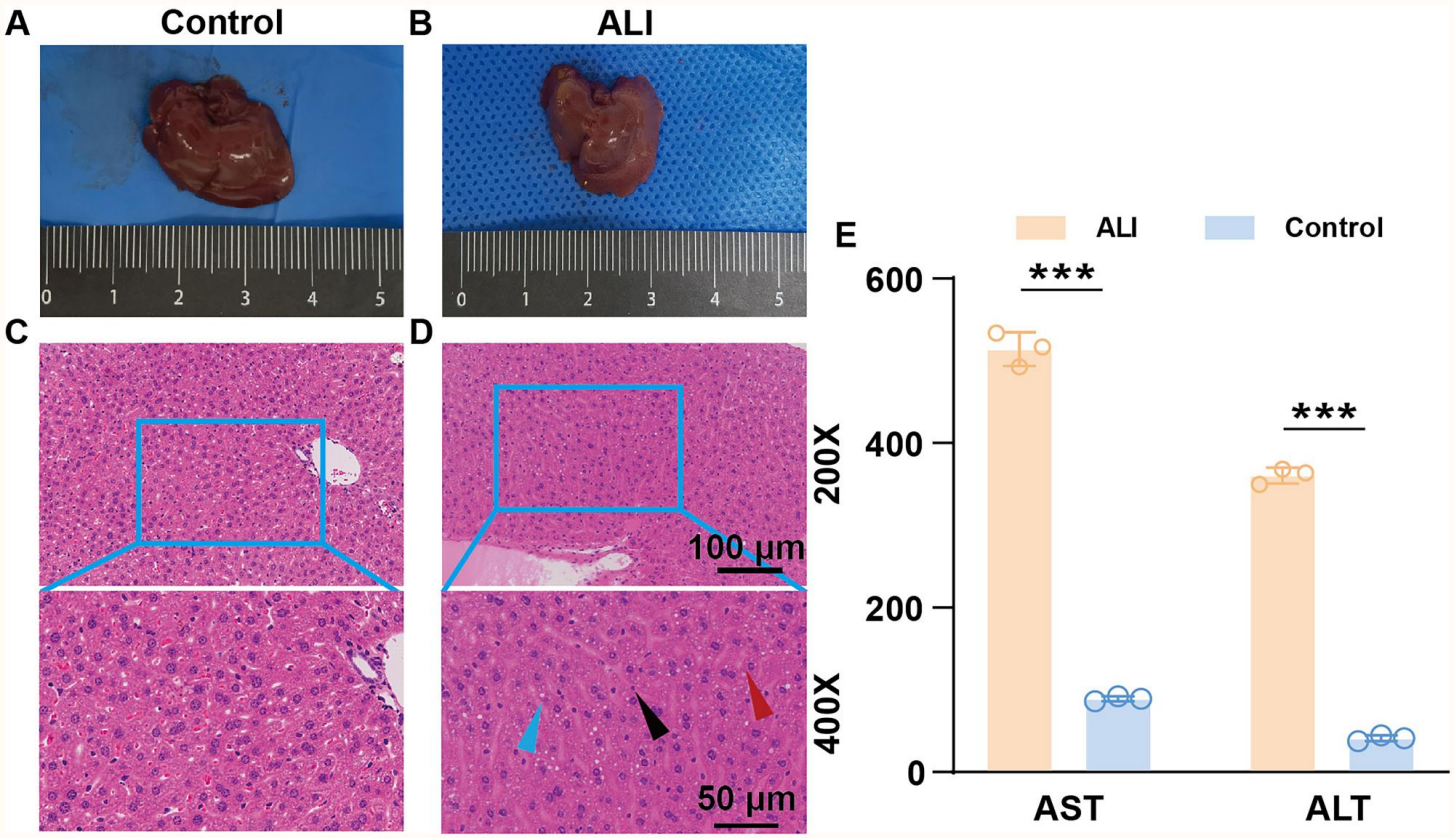
(**A**) Gross appearance of liver in control group. (**B**) Gross appearance of liver in ALI group. (**C**) HE staining of liver in control group. (**D**) HE staining of liver in ALI group (red arrow: macrovesicular steatosis; blue arrow: hydropic degeneration of hepatocytes; black arrow: inflammatory cells) (**E**). Serum AST and ALT levels in control and ALI group mice. All data are expressed as mean ± SD (*n* = 3). *t*-Test statistical analysis: *P < 0.05, **P < 0.01, ***P < 0.001, ns: no significance.

24 h after modeling, macroscopic observation of the liver revealed that compared with the normal liver, the liver in the model group showed a yellowish-brown color, tense capsule, blunt edge and rough surface (Figure 6A and B). HE staining showed disordered normal structure of hepatic lobules and loss of polarity in the arrangement of hepatic cell cords. Hepatocytes exhibited extensive hydropic degeneration, significantly increased volume, loose and light-stained cytoplasm and lipid droplet vacuoles of varying sizes. The hepatic sinusoidal space was significantly narrowed due to hepatocyte swelling, and numerous neutrophil infiltrations were observed (Figure 6C and D). Blood biochemical results showed that the levels of ALT and AST in the model group were significantly higher than those in the control group injected with olive oil solvent (Figure 6E). According to the authoritative identification of the chief physician of the Pathology Department of our hospital, the above changes were consistent with the manifestations of ALI.

**Figure 7.**
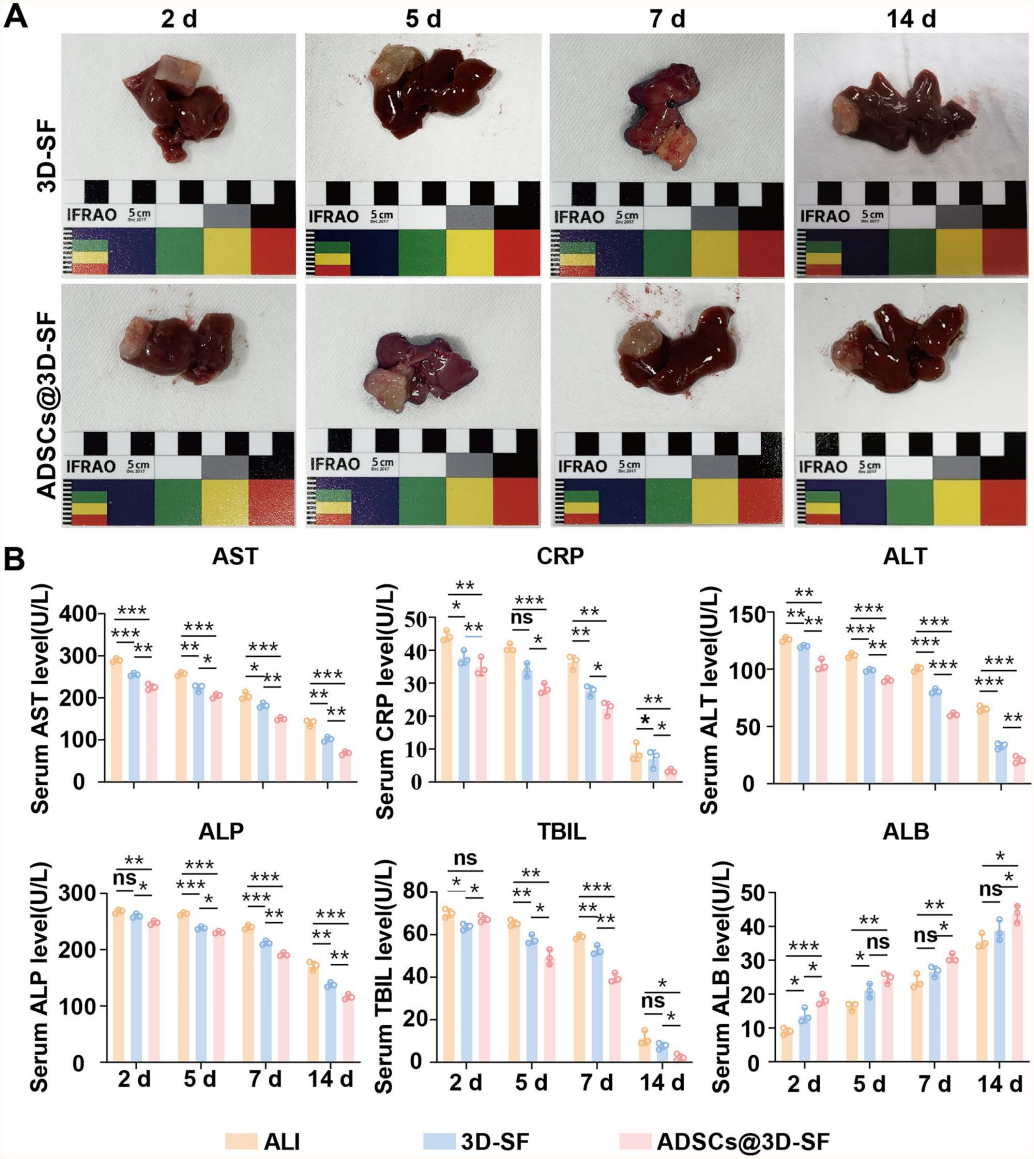
ADSCs@3D-SF Could promote the repair of ALI *in vivo*. (**A**) A general picture of liver repair after transplantation of 3D-SF and ADSCs@3D-SF. (**B**) The detection of biochemical indexes in mice serum, including ALT, AST, ALB, CRP, ALP and TBIL. All data are expressed as mean ± SD (*n* = 3). *t*-Test statistical analysis: *P < 0.05, **P < 0.01, ***P < 0.001, ns: no significance.

To observe ADSCs@3D-SF repair ALI in mice, we transplanted 3D-SF group and ADSCs@3D-SF group into ALI mice, respectively (Figure 7A). Within 14 days after transplantation, we observed that the boundary between the scaffold and liver tissue gradually disappeared, and tissue was visible on the surface of the scaffold. Furthermore, after transplantation, the scaffolds degraded gradually, with the ADSCs@3D-SF group showing a significantly faster degradation rate than the 3D-SF group (Figure S5).

Serum biochemical analyses were performed on days 2, 5, 7 and 14 post-transplantation to evaluate liver function recovery by assessing inflammatory and hepatic function markers. On day 2, serum levels of hepatocellular injury markers (AST, ALT), metabolic indicators (TBIL, ALP) and the inflammatory marker CRP were markedly elevated in all groups. However, both the 3D-SF and ADSCs@3D-SF groups showed consistently lower levels of these markers compared with the ALI group, with the most pronounced reduction observed in the ADSCs@3D-SF group. By day 14, indicators of liver injury and inflammation had decreased to their lowest levels following transplantation. In contrast, albumin, a marker of hepatic synthetic function, was initially reduced on day 2 but progressively increased over time, with significantly higher levels in the 3D-SF and especially the ADSCs@3D-SF groups compared with ALI. Similarly, TBIL and ALP levels remained lower in the 3D-SF and ADSCs@3D-SF groups throughout the experimental period, further declining with time but always below those of ALI (Figure 10). Collectively, these findings indicate that ADSCs@3D-SF transplantation more effectively attenuates hepatic inflammation, promotes tissue repair and facilitates recovery of liver function (Figure 7B). These results showed that ADSCs@3D-SF can effectively recover the liver injury according to biochemistry analysis.

### Histopathology analysis of 3D printed scaffolds repairing ALI of mice

**Figure 8.**
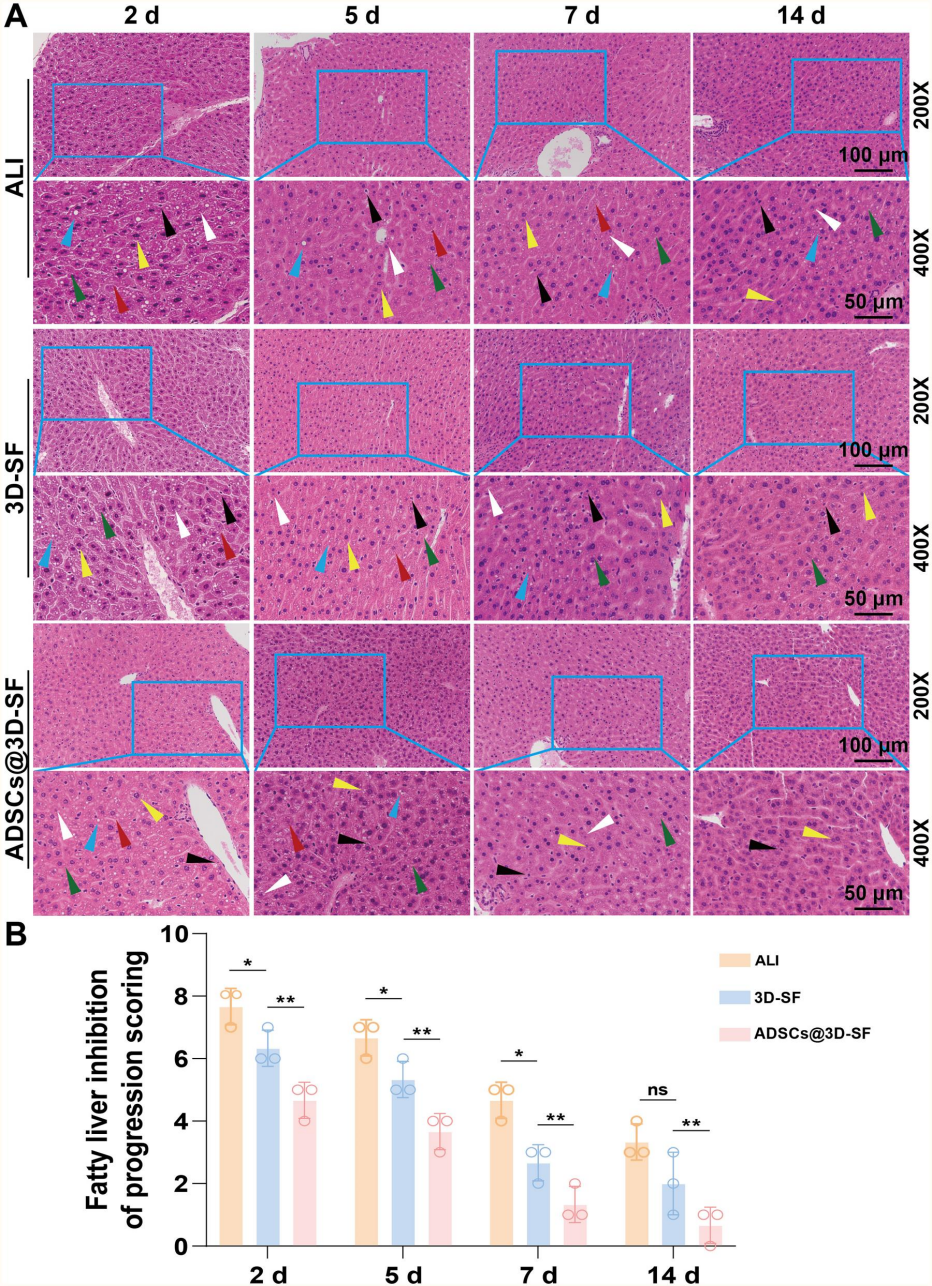
A histopathology analysis of 3D-SF repairing ALI of mice. (**A**) Histological analysis of liver in the three groups (ALI group, 3D-SF group, ADSCs@3D-SF group) by hematoxylin and eosin staining. (green arrow: vesicular steatosis; red arrow: hepatocellular hydropathy; yellow arrow: nuclear crumpling; black arrow: inflammatory cells; blue arrow: macrovesicular steatosis; white arrow: cytosolic nuclear degeneration). (**B**) Semi-quantitative analysis of H&E staining. All data are expressed as mean ± SD (*n* = 3). *t*-Test statistical analysis: *P < 0.05, **P < 0.01, ***P < 0.001, ns: no significance.

**Figure 9.**
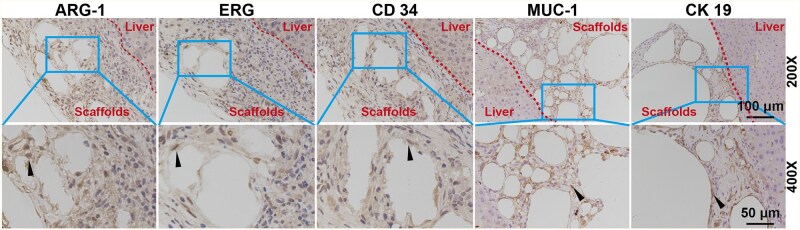
Formation of new tissue at the junction between the scaffold and the liver. Immunohistochemical staining of the damaged liver repair condition marker ARG-1, angiogenic markers CD34 and ERG, and bile duct markers MUC-1 and CK19 in ADSCs@3D-SF group. (black arrow: Positive intracellular protein expression of ARG 1, CD 34, ERG, MUC 1and CK 19 in ADSCs).

We found that all groups showed liver damage with hepatocellular hydropathy, hepatocellular steatosis, inflammatory cell infiltration and cytosolic degeneration after 2 days of implantation. Importantly, compared with the 3D-SF group, there were fewer macrovesicular steatosis in ADSCs@3D-SF group. On the fifth day of implantation, only a few macrovesicular steatosis were present in ADSCs@3D-SF group, but the lesions in the 3D-SF group mainly consisted of macrovesicular steatosis. After 14 days of implantation, the ADSCs@3D-SF group showed only a small number of inflammatory cells and hepatocyte hydropathy with no hepatocyte necrosis. However, the 3D-SF group presented a small amount of cytosolic nuclear degeneration that was still present (Figure 8A and B). These results confirmed that ADSCs@3D-SF could significantly reduce the inflammatory response in order to promote the repair of damaged liver in ALI (Figure 9). With the extension of the transplantation period, ADSCs@3D-SF integrated well with the liver surface and gradually degraded. To observe the newly generated tissues formed by ADSCs@3D-SF on the liver surface, we performed immunohistochemical staining of the newly generated tissues 2 weeks after transplantation. The results showed that angiogenic markers (ERG, CD34) and bile duct markers (MUC-1, CK19) were significantly expressed. Moreover, the liver repair marker ARG-1 was also significantly expressed in the newly formed tissues (Figure 8C).

### Transcriptomic analysis of newly formed tissues by 3D-PSFS

**Figure 10.**
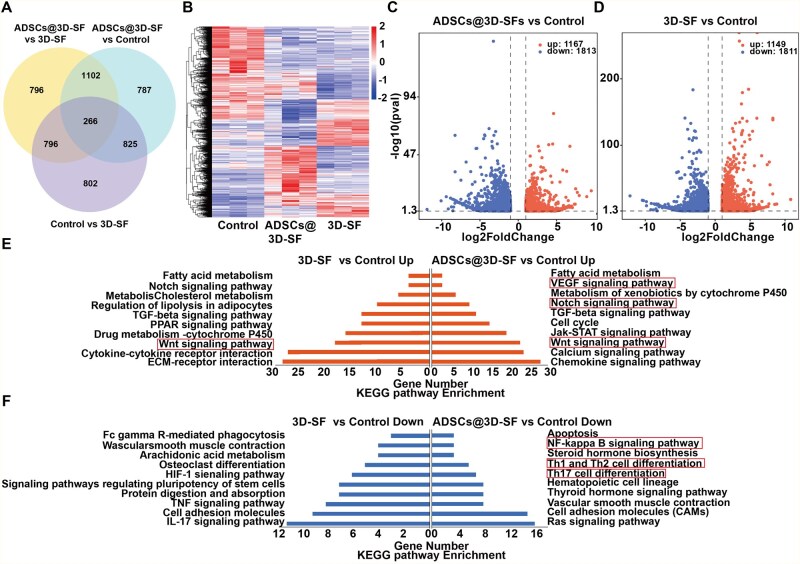
Analysis of RNA-sequences in the liver tissue-scaffold junction transplanted with 3D-SF and ADSCs@3D-SF. (**A**) Differentially expressed genes (DEGs) among groups in Venn diagram. (B) DEG expression clustering in heatmap. (**C, D**) DEG expression in volcano plot. (**E, F**) Analysis of KEGG pathway enrichment.

In order to investigate the potential mechanism of ADSCs@3D-SF for repairing damaged liver in mice, we performed transcriptome analysis of liver tissue-scaffold junction on the fifth day after transplantation. Venn diagram results showed that the 3D-SF group expressed 1889 differentially expressed genes, rather than the 1598 differentially expressed genes of ADSCs@3D-SF group (Figure 10A). Cluster analysis showed that the gene expression modules were similar in 3D-SF group and ADSCs@3D-SF group. However, they were significantly different from the ALI group (Figure 10B). Compared with the ALI group, the 3D-SF group indicated 1167 up-regulated genes and 1813 down-regulated genes, while ADSCs@3D-SF group expressed 1149 up-regulated genes, and 1811 down-regulated genes (Figure 10C and D). Moreover, KEGG enrichment analysis indicated many signaling pathways were altered in 3D-SF group and ADSCs@3D-SF compared with the ALI group, including upregulation of cell proliferation; hepatic drugs; lipids, fatty acid metabolism and inflammatory inhibitory pathways (Figure 10E). Importantly, there was a significant enrichment of Wnt signaling pathway, which plays an important character in the proliferation of cells. In addition, there were many immune response pathways such as Th1 and Th2 cell differentiation pathway; Th17 cell differentiation signaling pathway down-regulated both in ADSCs@3D-SF and 3D-SF group (Figure 10F).

### ADSCs loaded on 3D-PSFS upregulate the expression of proliferation pathway

**Figure 11.**
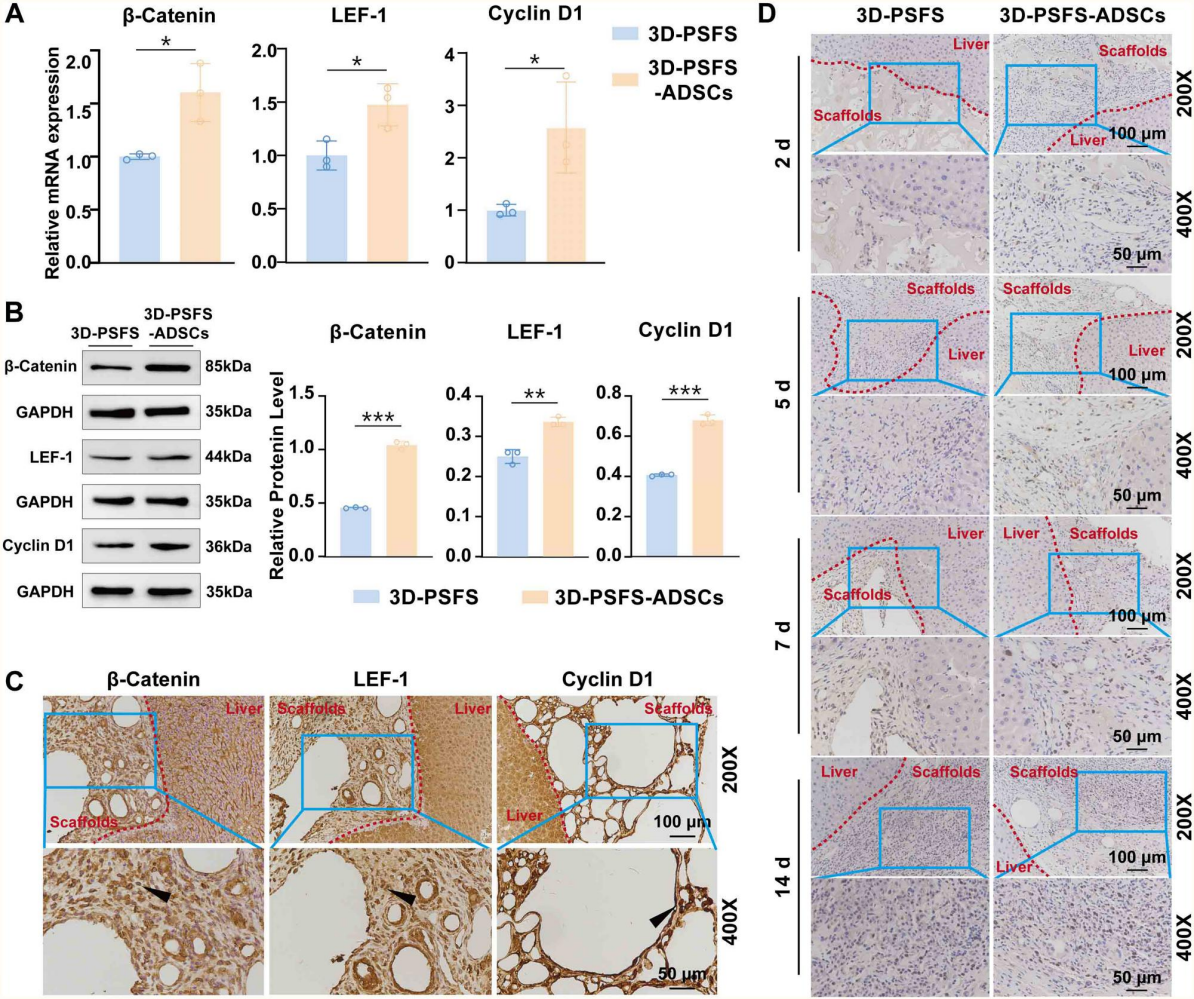
Wnt pathway of cells in cavity of ADSCs@3D-SF were highly activated in ALI mice. (**A**) The expression and quantitative analysis of β-catenin, LEF1 and cyclin D1 genes in 3D-SF and ADSCs@3D-SF group. (**B**) The expression and quantitative analysis of β-catenin, LEF-1 and cyclin D1 proteins in 3D-SF and ADSCs@3D-SF. (**C**) Immunohistochemical staining of β-catenin, LEF1 and cyclin D1 protein expression in ADSCs@3D-SF group. (black arrow: the positive expression of β-catenin, LEF 1 and cyclin D1 protein). (**D**) Immunohistochemical staining of Ki67 staining and semi-quantitative analysis of proliferation of different cells. All data were expressed as mean ± SD (*n* = 3). Statistical analysis: *P < 0.05, **P < 0.01, ***P < 0.001, ns: no significance.

To further validate the specific mechanism of repairing ALI after ADSCs@3D-SF, we used RT-qPCR, WB and immunohistochemical staining to assess the gene as well as protein expression in the Wnt signaling pathway in liver tissue-scaffold junction of the treated mice. The results of RT-qPCR (Figure 11A), and WB (Figure 11B) showed the β-Catenin, LEF1 and Cyclin D1 genes and protein expression in Wnt signaling pathway were significantly up-regulated in ADSCs@3D-SF treated liver tissue-scaffold junction compared with 3D-SF. Subsequently, immunohistochemical results (Figure 11C) showed that these three proteins’ expression levels were higher in ADSCs@3D-SF group than in 3D-SF group. In contrast to the 3D-SF group, there were plenty of positive cells stained by Ki67 in the junction area between scaffold of ADSCs@3D-SF and liver tissue. Moreover, the number of positive cells gradually showed an upward trend with the prolongation of transplantation time (Figure 11D). The results indicated that Wnt signaling pathway was involved in cell proliferation transplanted with ADSCs@3D-SF in ALI mice.

## Discussion

Despite the application of liver transplantation, bioartificial liver systems and stem cell therapy in the treatment of ALI, traditional approaches face challenges such as donor shortage, immune rejection and low cell survival rates. With the advancement of liver tissue engineering, therapeutic strategies involving biomaterial scaffolds to mimic the hepatic microenvironment and deliver stem cells for local transplantation have emerged. These approaches enhance stem cell engraftment within the liver, thereby exerting transdifferentiation and paracrine effects, ultimately improving treatment safety and clinical efficacy.

Among various biomaterial scaffolds, studies on the reparative effects and mechanisms of 3D-SF in ALI remain limited. Compared with conventional liver tissue engineering scaffolds, 3D-SF offers several advantages in repairing ALI: (i) The DLP-based 3D printing technique enables 3D-SF with high precision and resolution; (ii) 3D-SF exhibits a uniform architecture with a pore size of 100 μm, mechanical properties comparable to native liver tissue, and excellent biocompatibility with ADSCs; (iii) 3D-SF improves liver function and alleviates liver damage in ALI mice, and the combination of ADSCs with 3D-SF synergistically enhances the therapeutic efficacy; (iv) 3D-SF integrates well with host liver tissue, and vascular- and bile duct-like structures can form at the scaffold–liver interface; (v) ADSCs@3D-SF promotes cell proliferation and facilitates liver repair by upregulating the expression of β-catenin, LEF1, and Cyclin D1 at both the gene and protein levels in the Wnt signaling pathway.

Silk can be derived from multiple sources, including silkworms, spiders and other arthropods. Compared with SF obtained from other animals, Bombyx mori SF features a crystalline domain predominantly composed of β-sheet structures, conferring superior stability and ease of purification. These characteristics make it more suitable for 3D printing applications and capable of withstanding *in vivo* mechanical stress. Therefore, in this study, Bombyx mori silk was selected as the raw material for SF preparation and employed as the matrix for constructing 3D-SF scaffolds [25]. Currently, SF can be processed into films, fibers and sponges as biomaterial scaffolds; however, such scaffolds generally exhibit poor mechanical performance. Moreover, their microarchitecture typically consists of irregular porous cavities, which substantially limit ADSC adhesion and proliferation within the scaffold [26–30]. In contrast, DLP 3D printing, coupled with CAD, enables micro-scale design of scaffold architectures through layer-by-layer photopolymerization, allowing precise control of structural features [29, 31].

Methacrylation of SF is commonly achieved using monomers such as methacrylic anhydride (MA), 2-isocyanatoethyl methacrylate (IEM) and GMA. MA generates acidic by-products during the reaction, thereby interfering with SF crystallization and reducing efficiency. IEM enhances photocrosslinking but may increase the antigenicity of the material [32]. By contrast, GMA reacts with the amino groups of SF via epoxy ring-opening without generating by-products, offering greater controllability and superior biocompatibility [33]. Hence, in this study, GMA was employed for methacrylation modification to achieve an optimal balance between structural stability and biological functionality.

3D printing technologies provide precise control over scaffold parameters, which in turn modulate cellular attachment patterns and mechanosensing environments, ultimately influencing ADSCs proliferation and differentiation. Yan *et al.* [22] demonstrated that square-grid scaffold structures induced ADSCs to elongate along fiber orientations, facilitating cytoskeletal organization and accelerating proliferation. A pore size of 100 μm not only preserves scaffold stability but also ensures sufficient nutrient exchange and migration pathways for ADSCs, avoiding contact inhibition from overcrowding or compromised mechanics due to oversized pores. Such spatial configurations can further direct ADSCs lineage differentiation via specific mechanical cues, creating a favorable microenvironment for intercellular signaling [34, 35]. Mechanical properties also play a pivotal role in regulating ADSCs behavior. Studies by Wen, Bai, and colleagues [26, 36] revealed that when scaffold compressive moduli approximate those of soft tissues (5–15 kPa), integrin-mediated cell–matrix interactions promote the transmission of actomyosin contractility, thereby enhancing cell spreading, focal adhesion assembly and proliferation. This avoids scaffold softness (<1 kPa), which results in insufficient support and apoptosis, or excessive stiffness (>25 kPa), which suppresses proliferation through excessive mechanical stress. The compressive modulus of 3D-SF was measured at 12 kPa, closely matching that of ALI liver tissue (4–10 kPa), thereby minimizing stress-induced injury and providing an ideal microenvironment for proliferation [37, 38]. In terms of differentiation, scaffold elasticity exerts a decisive influence on ADSCs lineage specification: soft matrices (∼1 kPa) favor neuronal differentiation, intermediate stiffness (∼10 kPa) promotes myogenic differentiation, whereas rigid substrates (∼100 kPa) bias osteogenic differentiation [38]. The 3D-SF scaffolds developed in this study exhibited a compressive modulus comparable to that of ALI liver tissue, effectively inducing ADSCs differentiation toward hepatocyte-like cells, accompanied by significant upregulation of liver-specific markers such as ALB and CYP1A1 [32, 39, 40]. Taken together, scaffold architecture and stiffness act synergistically through a ‘spatial-mechanical’ effect to regulate ADSCs behavior, ultimately influencing both proliferation and differentiation.

Following transplantation of ADSCs@3D-SF into ALI mice, significant improvements in liver function were observed within 2 weeks. At the interface between the implanted scaffold and host liver tissue, newly formed vascular and bile duct-like structures were detected. Transcriptomic analysis, RT-qPCR, WB and histological examinations consistently demonstrated cytoplasmic accumulation of β-catenin accompanied by upregulation of LEF1 and Cyclin D1, indicating activation of the Wnt/β-catenin–LEF1 axis. This activation was associated with cell cycle progression and initiation of regeneration-related gene programs, thereby supporting liver tissue reconstruction. Moreover, ADSCs synergistically enhanced the reparative effect of 3D-SF on liver injury in ALI models [41–43]. These findings provide evidence that a coordinated ‘material–cell–signal’ interaction underlies liver regeneration. Nevertheless, the precise mechanistic role of this signaling pathway in ADSCs proliferation remains to be fully elucidated in future studies.

The *in vivo* inflammatory milieu plays a decisive role in determining scaffold-mediated repair outcomes, primarily through regulation of macrophage M1/M2 polarization and T cell responses [44, 45]. Transcriptomic analysis in this study revealed that, compared with the ALI group, both the 3D-SF and ADSCs@3D-SF groups exhibited significant downregulation of genes involved in the IL-17 signaling pathway and Th1, Th2 and Th17 cell differentiation, suggesting attenuation of hepatic inflammatory responses. Previous studies have shown that ADSCs can activate Arg 1 expression in macrophages via exogenous STAT3 signaling, thereby inducing their transition to an anti-inflammatory M2 phenotype [46], while M2 macrophages in turn promote Th2-type immune responses [47]. Although our study included ADSCs, hepatocytes and multiple immune cell types, the specific immunosuppressive role of Arg 1 requires further validation. Future work should incorporate immune cell profiling and quantitative analyses to clarify the cellular interactions and signaling mechanisms through which 3D-SF scaffolds mitigate hepatic inflammation.

In addition, this therapeutic strategy holds potential clinical value. For patients with acute liver failure caused by drug-induced injury or viral hepatitis, laparoscopic scaffold transplantation could serve as a bridging therapy, providing temporary functional support while awaiting donor availability without imposing an additional burden on the liver. In cases of extensive hepatectomy for liver tumors, scaffold transplantation may promote regeneration and thereby prevent postoperative liver failure. Although animal experiments cannot fully replicate the complex pathology of human acute liver failure, this study offers mechanistic evidence for scaffold–stem cell synergistic repair and provides new directions for clinical translation.

## Conclusions

In general, the experimental findings offer a new insight in the application of 3D-SF to improve the effectiveness of ADSCs transplantation for treating ALI. 3D-SF showed the desired biocompatibility, biostability and mechanical properties in the proliferation of ADSCs. Notably, this study is the first application of 3D-SF to induce ADSCs into hepatic-like cells *in vitro* and combine 3D-SF with ADSCs for the treatment of ALI. Based on these results, the 3D-SF may become a promising scaffold for the therapy of ALI in clinical applications.

## Supplementary Material

rbaf103_Supplementary_Data

## Data Availability

Data supporting the results of this study can be obtained from the corresponding author upon reasonable request.

## Management of experimental sites and facilities

Jiang Peng, Institute of Orthopedics, Beijing Key Lab of Regenerative Medicine in Orthopedics, Key Laboratory of Musculoskeletal Trauma & War Injuries PLA, Chinese PLA General Hospital, Beijing 100853, PR China.
