# Supplementary material for: Tissue-engineered liver using 3D-printed silk fibroin scaffolds loaded with stem cells for the treatment of acute liver injury
Source: Regen Biomater. 2025 Oct 23;12:rbaf103. doi: 10.1093/rb/rbaf103 (PMC12639544; doi:10.1093/rb/rbaf103)
Supplement: rbaf103_Supplementary_Data [file rbaf103_supplementary_data.zip › Fig S2.docx]

**^1^H NMR Chemical Shifts and Integrals of Sil-MA and SF**

| **Sil-MA** **spectrum** | **Chemical Shift, δ** | **Integral** |
| --- | --- | --- |
| -CH3 | 1.23 ppm | 12 |
| Lysine | 2.83 ppm | 0.26 |
| =CH2 | 7.04 – 6.38 ppm | 8 |
| **SF** **spectrum** | **Chemical Shift, δ** | **Integral** |
| -CH3 | 1.23 | 9 |
| Lysine | 2.82 | 0.63 |
