# Supplementary material for: Tissue-engineered liver using 3D-printed silk fibroin scaffolds loaded with stem cells for the treatment of acute liver injury
Source: Regen Biomater. 2025 Oct 23;12:rbaf103. doi: 10.1093/rb/rbaf103 (PMC12639544; doi:10.1093/rb/rbaf103)
Supplement: rbaf103_Supplementary_Data [file rbaf103_supplementary_data.zip › Fig. S6.docx]

**Table 1. Molecules that promote repair/activate Wnt signaling pathway**

| **Molecule** | **Category** | **Major Function** | **Mechanistic Notes** |
| --- | --- | --- | --- |
| Nkd1, Nkd2 | Negative feedback regulators | Enhance hepatocyte proliferative capacity and repair through fine-tuned feedback regulation | Upon Wnt ligand-dependent activation, the pathway is reciprocally inhibited, thereby stabilizing cell proliferation–related signaling. [18] |
| CCN4 (WISP1) | Downstream effector/matrix factor | Accelerates hepatocyte cell cycle progression,promotes proliferation and migration | Facilitates cell cycle progression and migration; participates in post-injury tissue remodeling [19] |
| GPC4, LRP5, LGR5 | Coreceptors/stem cell markers | Activate Wnt/β-catenin signaling; promote hepatic stem/progenitor cell proliferation and differentiation | Cooperate with FZD complexes to amplify canonical Wnt signaling; LGR5(+) stem cells expand under Wnt stimulation [20–22] |
| FZD1/2/4/5 | Receptors | Initiate Wnt signaling, regulate proliferation, anti-apoptosis, migration, and tissue remodeling | FZD-LRP complexes mediate β-catenin signaling and noncanonical branches [23] |
| RAC2 | Small GTPase | Remodels cytoskeleton, promotes migration toward injury sites | Regulates F-actin and adhesion dynamics, enhances directed migration [24] |
| RSPO1, RSPO3 | Amplifiers | Potentiate Wnt/β-catenin signaling, promote stem cell proliferation and regeneration | Amplify receptor availability and signaling strength via the LGR–ZNRF3/RNF43 axis [20] |
| PORCN | Post-translational modification enzyme | Maintains Wnt ligand lipidation and secretion activity, promotes pathway activation | Essential for Wnt O-acylation, ensuring ligand stability and functionality [25] |
| WNT10B | Ligand | Enhances Wnt/β-catenin activation, promotes regeneration | Upstream ligand signal upregulation drives proliferation-associated transcription [26] |

**Table 2. Molecules mediating negative feedback/braking Wnt signaling pathway to prevent excessive proliferation and fibrosis**

| **Molecule** | **Category** | **Major Function** | **Mechanistic Notes** |
| --- | --- | --- | --- |
| SFRP1, SFRP2 | Secreted antagonists | Prevent Wnt overactivation, inhibit fibrosis, ensure proper repair | Bind Wnt ligands/receptors, reducing pathway intensity [22] |
| DKK2 | Secreted antagonist/regulator | Suppresses Wnt overactivation, prevents fibrogenesis | Interferes with the availability of LRP5/6 receptors, restricting the pathway. [27] |
| AXIN2 | Negative feedback target/scaffold protein | The pathway’s intrinsic negative feedback mechanism preserves the balance between regeneration and homeostasis | Forms degradation complex to limit β-catenin signaling [28] |

**Table 3. Alterations in scaffold degradation-related pathways in the ADSCs@3D-SF group**

| **Signaling pathway** | **Regulation** | **Main mechanism** | **Effect on scaffold degradation** |
| --- | --- | --- | --- |
| Jak-STAT signaling pathway | Upregulated | Enhances ADSC metabolic activity → increased acidic metabolites; elevated ROS generation | Reduced local pH promotes silk fibroin hydrolysis; ROS oxidize crosslinked structures, accelerating degradation [40–43] |
| Chemokine signaling pathway | Upregulated | ADSCs secrete chemokines to recruit macrophages and neutrophils | Macrophages phagocytose degradation fragments; release of MMPs and other enzymes directly degrades silk fibroin [44, 45] |
| Apoptosis signaling pathway | Downregulated | Suppresses excessive apoptosis | Preserves immune cell function, enabling their continuous participation in scaffold degradation [46] |
| NF-κB signaling pathway | Downregulated | Inhibits excessive inflammatory responses | Maintains immune cell stability, promoting sustained degradation [47] |
| Fatty acid metabolism signaling pathway | Upregulated | Enhances energy metabolism | Provides the energy for secretion of degradation-related enzymes and factors [48] |
| VEGF signaling pathway | Upregulated | Promotes angiogenesis | Enhances blood perfusion and fluid exchange, facilitating enzyme delivery and degradation product clearance, thus accelerating scaffold degradation [49, 50] |

**Table 4-1 Functional Roles of Genes in the Th17 Cell Differentiation Pathway**

| **Gene** | **Mechanism of Action** | **Effect on ALI Repair** |
| --- | --- | --- |
| Il2rb | Suppresses excessive Th17 activation via a dual regulatory mechanism while promoting Treg proliferation to maintain immune balance | Prevents uncontrolled inflammatory response, enhances immunosuppression, alleviates liver inflammation, and promotes repair [58, 59] |
| Gata3 | Antagonizes RORγt expression, inhibits Th17 differentiation, shifts immune response toward Th2 phenotype, and exerts anti-inflammatory effects | Reduces pro-inflammatory cytokine levels, promotes anti-inflammatory cytokine secretion, and creates an anti-inflammatory microenvironment conducive to hepatocyte regeneration [60–62] |
| Mapk13 | Modulates STAT3 phosphorylation levels to precisely control the effector function of Th17 cells | Maintains appropriate inflammation balance, facilitating clearance of necrotic cells while avoiding excessive inflammation and parenchymal damage [63–65] |
| Hsp90aa1 | Stabilizes IκBα and inhibits the NF-κB signaling pathway, preventing cytokine storm | Reduces risk of cytokine storm and protects hepatocytes from secondary damage [66, 67] |
| H2-DMa | Regulates antigen presentation and influences T-cell activation threshold to ensure a moderated immune response | Lowers risk of excessive immune activation, ensures appropriate immune response, and reduces hepatocyte necrosis [68] |
| Cd3e | Modulates TCR signal strength to ensure immune response is neither excessive nor insufficient | Ensures effective clearance of necrotic tissue without excessive immune attack, thereby accelerating repair [69] |

**Table 4-2 Functional Roles of Genes in the Th1 and Th2 Cell Differentiation Pathway**

| **Gene** | **Mechanism of Action** | **Effect on ALI Repair** |
| --- | --- | --- |
| H2DMa | Acts as an MHC II chaperone to regulate antigen presentation | Prevents excessive T-cell activation and reduces secondary immune injury to liver tissue [70] |
| Gata3 | Drives Th2 differentiation and promotes secretion of IL-4/IL-5/IL-13 | Promotes anti-inflammatory response and tissue repair, facilitating hepatocyte regeneration [61] |
| Il2rb | Mediates IL-2 signaling and maintains Treg function | Balances Th1/Th2 immunity, suppresses excessive inflammation, and supports repair [71] |
| Cd3e | Key component of the TCR complex; modulates TCR signaling | Ensures appropriate immune activation and avoids persistent inflammation leading to liver injury [69] |
| Mapk13 | Phosphorylates STAT4 to regulate Th1 function | Fine-tunes inflammatory response and prevents excessive Th1 activity from causing hepatocyte necrosis [72] |
