# Supplementary figures and images for: Tissue-engineered liver using 3D-printed silk fibroin scaffolds loaded with stem cells for the treatment of acute liver injury
Source: Regen Biomater. 2025 Oct 23;12:rbaf103. doi: 10.1093/rb/rbaf103 (PMC12639544; doi:10.1093/rb/rbaf103)

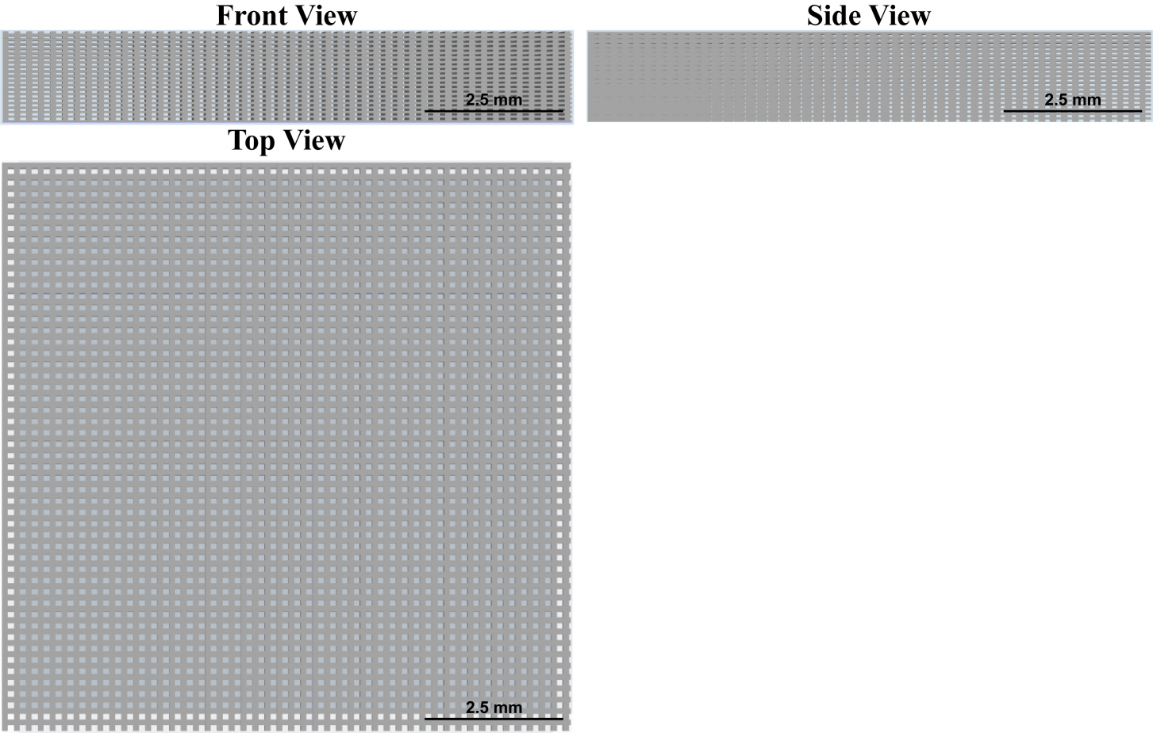

Supplement: rbaf103_Supplementary_Data [file rbaf103_supplementary_data.zip › Fig S3.jpg]

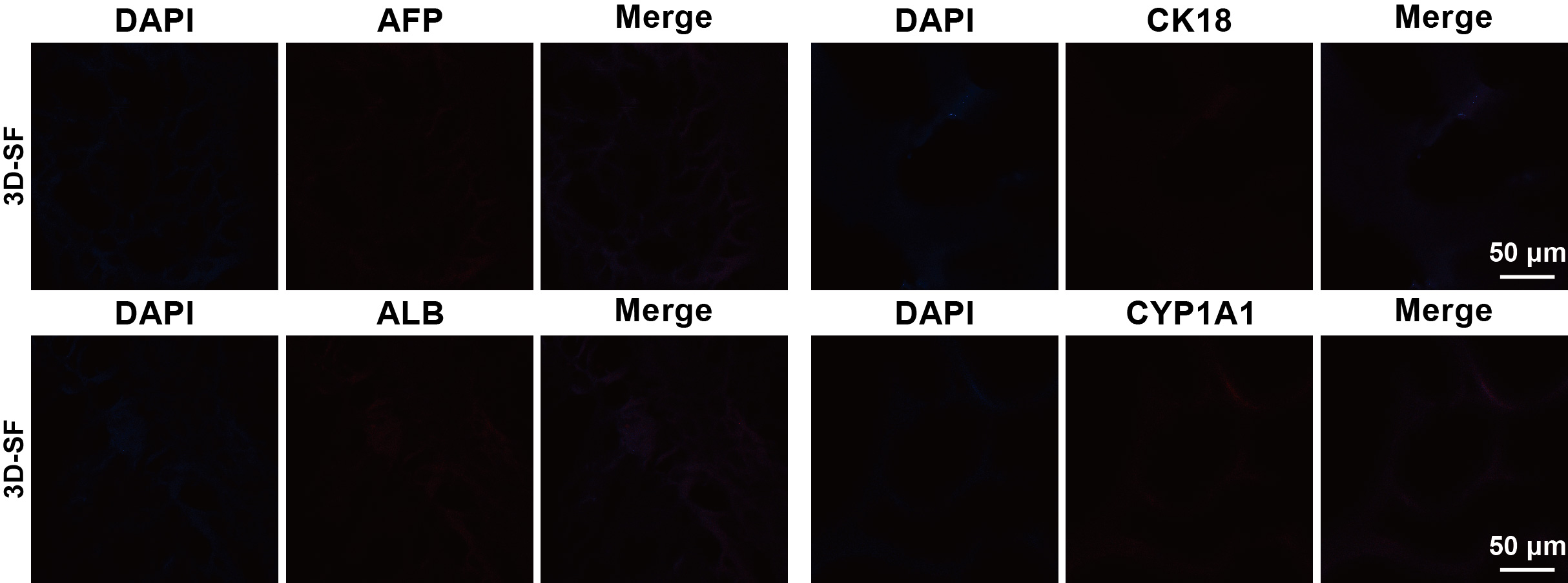

Supplement: rbaf103_Supplementary_Data [file rbaf103_supplementary_data.zip › Fig.S4.jpg]

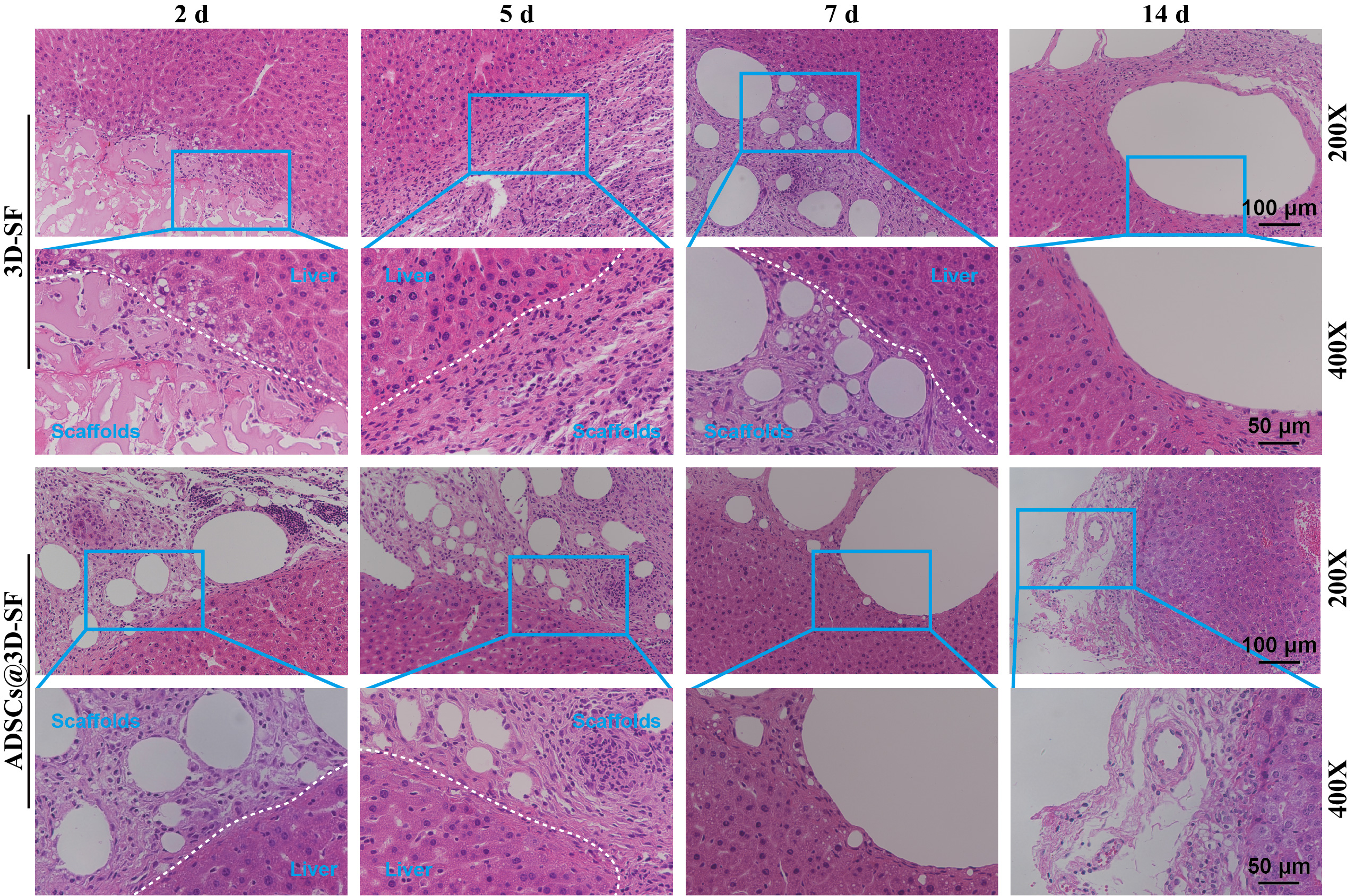

Supplement: rbaf103_Supplementary_Data [file rbaf103_supplementary_data.zip › Fig.S5.jpg]
